# Molecular mechanism of ER stress-induced pre-emptive quality control involving association of the translocon, Derlin-1, and HRD1

**DOI:** 10.1038/s41598-018-25724-x

**Published:** 2018-05-09

**Authors:** Hisae Kadowaki, Pasjan Satrimafitrah, Yasunari Takami, Hideki Nishitoh

**Affiliations:** 1 Laboratory of Biochemistry and Molecular Biology, Department of Medical Sciences, University of Miyazaki, 5200 Kihara, Kiyotake, Miyazaki, 889-1692 Japan; 2 Department of Chemistry, Faculty of Sciences, Tadulako University, Kampus Bumi Tadulako Tondo, Palu, 94118 Indonesia

## Abstract

The maintenance of endoplasmic reticulum (ER) homeostasis is essential for cell function. ER stress-induced pre-emptive quality control (ERpQC) helps alleviate the burden to a stressed ER by limiting further protein loading. We have previously reported the mechanisms of ERpQC, which includes a rerouting step and a degradation step. Under ER stress conditions, Derlin family proteins (Derlins), which are components of ER-associated degradation, reroute specific ER-targeting proteins to the cytosol. Newly synthesized rerouted polypeptides are degraded via the cytosolic chaperone Bag6 and the AAA-ATPase p97 in the ubiquitin-proteasome system. However, the mechanisms by which ER-targeting proteins are rerouted from the ER translocation pathway to the cytosolic degradation pathway and how the E3 ligase ubiquitinates ERpQC substrates remain unclear. Here, we show that ERpQC substrates are captured by the carboxyl-terminus region of Derlin-1 and ubiquitinated by the HRD1 E3 ubiquitin ligase prior to degradation. Moreover, HRD1 forms a large ERpQC-related complex composed of Sec61α and Derlin-1 during ER stress. These findings indicate that the association of the degradation factor HRD1 with the translocon and the rerouting factor Derlin-1 may be necessary for the smooth and effective clearance of ERpQC substrates.

## Introduction

In co-translational translocation, secretory and transmembrane proteins possessing signal sequences or transmembrane domains are targeted to the ER membrane and translocated into the ER via the docking of a ribosome to the translocon. Translocated nascent polypeptides are folded correctly and secreted or transported to the membrane compartment. The disturbance of ER homeostasis leads to an accumulation of unfolded or misfolded proteins and causes ER stress, which disrupts ER function. Cells resolve ER stress through the unfolded protein response (UPR), which is triggered by the three ER transmembrane receptors PERK, ATF6 and IRE1 and mediates the following four well-known quality control systems: (1) translational attenuation; (2) the selective degradation of ER-associated mRNAs; (3) ER-associated degradation (ERAD), by which unfolded or misfolded proteins are retrotranslocated from the ER into the cytosol and degraded by the ubiquitin-proteasome system (UPS); and (4) the transcriptional activation of genes encoding factors related to ER protein folding and ERAD. These systems can be classified into two strategies: limiting further protein loading into the ER, as in (1) and (2), and restoring folding capacity in the ER via the induction of ER chaperones and the clearance of unfolded or misfolded proteins, as in (3) and (4). In the former strategy, pre-emptive quality control (pQC) serves to degrade ER-targeting proteins specifically by translocational attenuation during ER stress^1,2^. ER stress-induced pQC (ERpQC) is thought to occur as follows^1,3,4^: (1) in co-translational translocation, the hydrophobic signal peptide of nascent polypeptides that emerges from the ribosome is recognized by the signal recognition particle (SRP); (2) the ribosome nascent chain (RNC)-SRP complex is targeted to the translocon; (3) the translocation of specific ER proteins is attenuated by an unknown mechanism during ER stress; (4) translationally and translocationally attenuated nascent chains are released from the translocon; and (5) the fully translated proteins in the cytosol are degraded by the UPS. This pre-emptive degradation system restricts excessive protein loading into the ER and protects cells against ER stress. We have previously investigated the mechanisms of ERpQC, which includes a rerouting step and a degradation step^5^. In the rerouting step, a specific newly synthesized polypeptide on the translationally arrested RNC-SRP complex is rerouted from the ER translocation pathway to the cytosolic degradation pathway without the cleavage of its signal peptide. This rerouting step is triggered by the interaction of SRP with Derlin family proteins (Derlins; Derlin-1, Derlin-2 and Derlin-3 in mammals), which are components of the ERAD complex and play a role in the retrotranslocation of unfolded or misfolded proteins^6–8^. In the degradation step, fully translated polypeptides are ubiquitinated by an unknown E3 ligase, transported to the proteasome by the p97 AAA ATPase and the chaperone Bag6, and finally degraded by the proteasome. In ERAD, p97 and Bag6 cooperate in the retrograde transport of unfolded or misfolded proteins from the ER to the cytosol and the maintenance of these retrotranslocated proteins in soluble states through holdase activity, respectively. Recently, it has been revealed that Bag6 recognizes hydrophobic segments of ERpQC substrates, and the ubiquitin-associated ER membrane protein arsenite-inducible RNA-associated protein-like (AIRAPL) directly interacts with the ubiquitinated ERpQC substrates and transfers them to p97, which is followed by proteasomal degradation^9^. The disturbance of ERpQC leads to conformational diseases caused by the aggregation of mislocalized prion proteins^10^. Although the ERpQC pathway might be a therapeutic target for some conformational diseases, the molecular mechanisms of ERpQC are not fully understood. Importantly, the mechanisms by which ER-targeting proteins are selectively rerouted from translocation to degradation and which E3 ligase targets ERpQC substrates remain to be clarified.

Numerous E3 ligases have been reported to contribute to the ubiquitination of ERAD substrates, mislocalized proteins, and nascent polypeptide chains in ribosome-associated quality control to maintain proteostasis. In the mammalian ERAD system, the well-known E3 ligases HRD1, gp78, RMA1/RNF5, and TEB4 are embedded in the ER membrane and form a complex with other factors involved in substrate recognition, recruitment to the retrotranslocon, retrotranslocation, extraction, and transport to the proteasome^11,12^. A distinct ERAD complex, including different E3 ligases, defines the degradation of several types of substrates. Moreover, other ERAD-related E3 ligases, including TRC8/RNF139, RNF170, RNF103, and RFP2/TRIM13, are also located on the ER transmembrane^11,12^. Mislocalized membrane proteins or secretory proteins are targeted for disposal by the UPS to avoid the aggregation and perturbation of cytosolic protein homeostasis^13,14^. Mislocalized cytosolic proteins are protected from aggregation by the Bag6 complex and ubiquitinated by the Bag6-associated E3 ligase RNF126^13,14^. Stalled ribosomes due to aberrant translation are recognized and split by the translation factor complex Pelota-Hbs1^15,16^. In this pathway, nascent polypeptide chains on the 60 S ribosomal subunit are ubiquitinated by the cytosolic E3 ligase Listerin, extracted by p97, and finally degraded by the proteasome^17–19^. These E3 ligases are candidates that might contribute to the ubiquitination of cytosolic-rerouted ER-targeting proteins.

Here, we show that the carboxyl-terminus region (CT) of Derlin-1 is indispensable for the rerouting of ERpQC substrates, and the well-known ERAD-related E3 HRD1 is required for the degradation of ERpQC substrates through its E3 ligase activity. HRD1 interacts with ERpQC substrates and forms a large ERpQC-related complex composed of the translocon component Sec61 and the rerouting factor Derlin-1 during ER stress. Association of the translocon, Derlin-1, and HRD1 may compose a hub of the ERpQC machinery.

## Results

### The carboxyl-terminus region of Derlin-1 is required for the rerouting of ERpQC substrates

**Figure 1:**
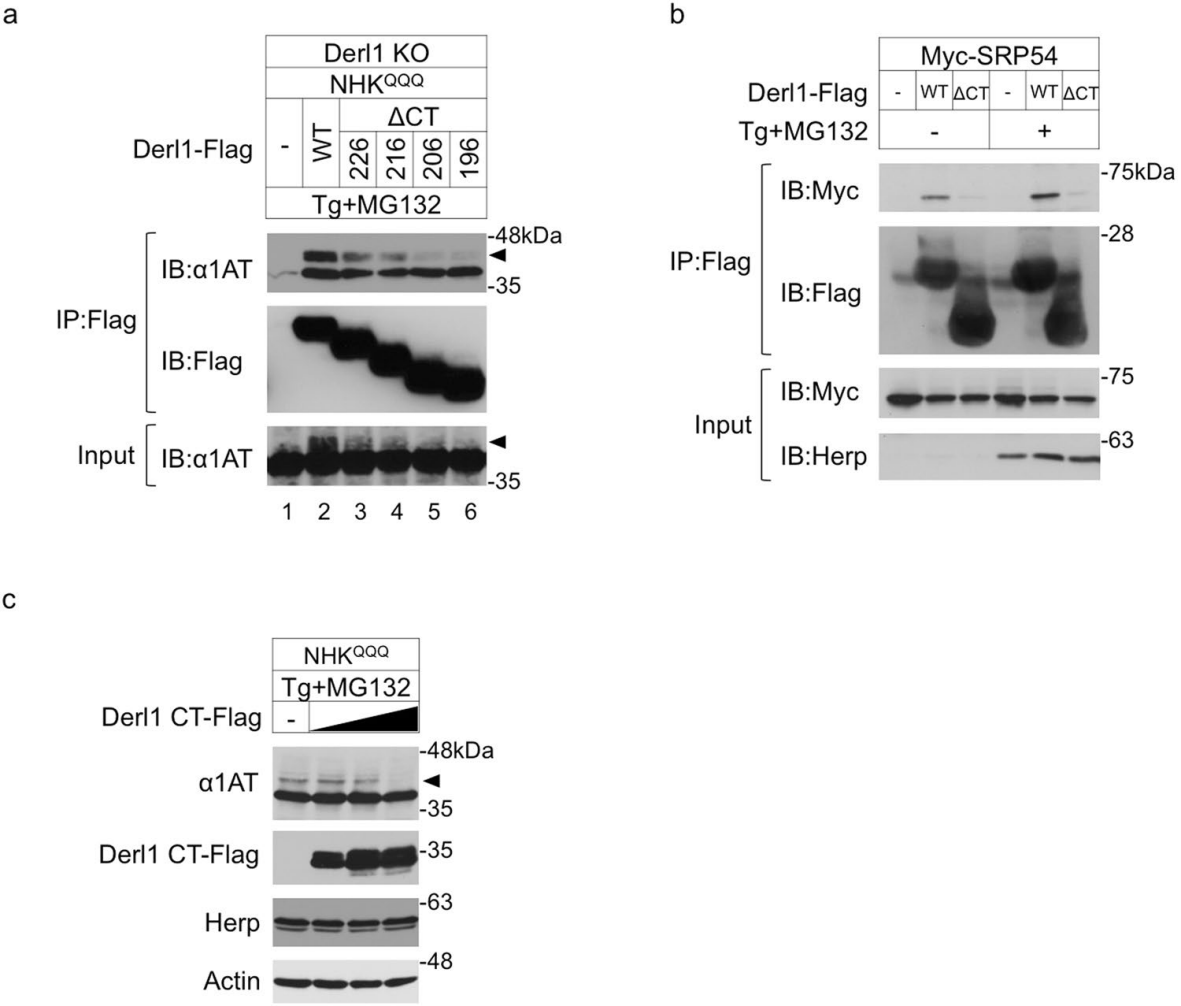
The carboxyl-terminus region of Derlin-1 is required for the rerouting of ER pQC substrates. (**a**) The requirement of Derlin-1 carboxyl-terminus region (CT) for the Derlin-1-mediated rerouting of ERpQC substrates. Derlin-1 knockout HEK293 cells (Derl1 KO) were transfected with NHK^QQQ^ and Derlin-1-Flag [wild-type (WT) or C-terminal-truncated mutant form (ΔCT)] and treated with 50 nM thapsigargin (Tg) and 200 nM MG132 for 16 h. Derlin-1-Flag was immunoprecipitated (IP) with an anti-Flag antibody (Ab) affinity gel and analyzed by immunoblotting (IB) with the indicated Abs. Arrowheads, signal peptide-uncleaved NHK^QQQ^ (^S^NHK^QQQ^); ΔCT226, Derlin-1 (a.a. 1–226); ΔCT216, Derlin-1 (a.a. 1–216); ΔCT206, Derlin-1 (a.a. 1–206); ΔCT196, Derlin-1 (a.a. 1–196) (Supplementary Fig. 1b). (**b**) The requirement of Derlin-1 CT for the interaction between Derlin-1 and SRP54. HEK293 cells transfected with Myc-SRP54 and Derlin-1-Flag WT or ΔCT196 were treated with 50 nM Tg and 200 nM MG132. The lysates from HEK293 cells were analyzed by IP-IB. ΔCT, Derlin-1 (a.a. 1–196). (**c**) Inhibition of the accumulation of ERpQC substrates by Derlin-1 CT. HEK293 cells were transfected with Venus-Derlin-1 CT-Flag in a gradually changed amount of plasmid and NHK^QQQ^ and treated with 50 nM Tg and 200 nM MG132. Cell lysates were analyzed by IB. Arrowhead indicates ^S^NHK^QQQ^. Derl1 CT-Flag, Derlin-1 (a.a. 197–251). (**a**–**c**) Full-length blots are presented in Supplementary Fig. 5.

We previously studied the mechanism of ERpQC, which controls protein loading into the ER through Derlin-mediated rerouting from the ER to the cytosol prior to proteasomal degradation^5^. The null Hong Kong (NHK) mutant of α1-antitrypsin lacking N-glycosylation sites (NHK^QQQ^) was used as the ERpQC substrate as previously reported^5^. In wild-type (WT) HEK293 cells treated with the ER stressor thapsigargin (Tg) and a proteasome inhibitor (MG132), the accumulation of signal peptide-uncleaved NHK^QQQ^ (^S^NHK^QQQ^, an ERpQC substrate) was observed (Supplementary Fig. 1a, top panel, lane 3, arrowhead), but this form was absent from Derlin-1 knockout (Derl1 KO) HEK 293 cells (Supplementary Fig. 1a, top panel, lane 7), consistent with our previous study^5^. Exogenously expressed Derlin-1 WT reconstituted this phenotype in Derl1 KO cells (Supplementary Fig. 1a, top panel, lane 11). Notably, cells overexpressing Derlin-1 showed increased expression of ERpQC substrates (Supplementary Fig. 1a, lanes 9–12). These observations support our previous findings that Derlin-1 positively reroutes ERpQC substrates prior to proteasomal degradation during ER stress^5^. We then investigated which regions of Derlin-1 are required for rerouting activity. Derlin-1 has six predicted transmembrane (TM) domains and cytosolic-facing amino (N)- and carboxyl (C)-termini^20^ (Supplementary Fig. 1b). Because ERpQC substrates are rerouted to the cytosol and the cytosolic-facing N-terminal region of Derlin-1 is short, we constructed expression plasmids for C-terminal-truncated Derlin-1 proteins [Derlin-1 (ΔCT)] that included all TM domains (Supplementary Fig. 1b) to identify the essential domain for the capture and rerouting of ERpQC substrates. Deletion of the CT (a.a. 207–251 or a.a. 197–251) clearly abolished the Derlin-1-mediated accumulation of ERpQC substrates and the interaction of Derlin-1 with these substrates (Fig. 1a, input and top panels, lanes 5 and 6, arrowheads). These findings suggest that the CT is required for the Derlin-1-mediated rerouting of ERpQC substrates. Derlins are coupled with the 54-kDa subunit of the SRP (SRP54)-SRP receptor (SR) complex to reroute ERpQC substrates from the translocation pathway to the degradation pathway during ER stress^5^. We therefore examined whether the CT of Derlin-1 is required for interaction with SRP54. ER stress enhanced the interaction of Derlin-1 WT with SRP54, and the deletion of the CT attenuated its binding affinity (Fig. 1b). Based on these findings, which suggest that Derlin-1 reroutes ER-targeting proteins from the translocation pathway to the cytosolic degradation pathway by interacting with the SRP-SR complex, we hypothesized that forcing the dissociation of Derlin-1 from SRP54 would attenuate the rerouting of ERpQC substrates during ER stress. We therefore evaluated the dominant negative effect of the Derlin-1 CT on the endogenous Derlin-1-mediated rerouting of ERpQC substrates. As expected, overexpressing Derlin-1 CT inhibited the ER stress-induced accumulation of ^S^NHK^QQQ^ (Fig. 1c, top panel). These observations suggest that the Derlin-1 CT plays a pivotal role in rerouting ERpQC substrates.

### HRD1 is required for the degradation of ERpQC substrates

**Figure 2:**
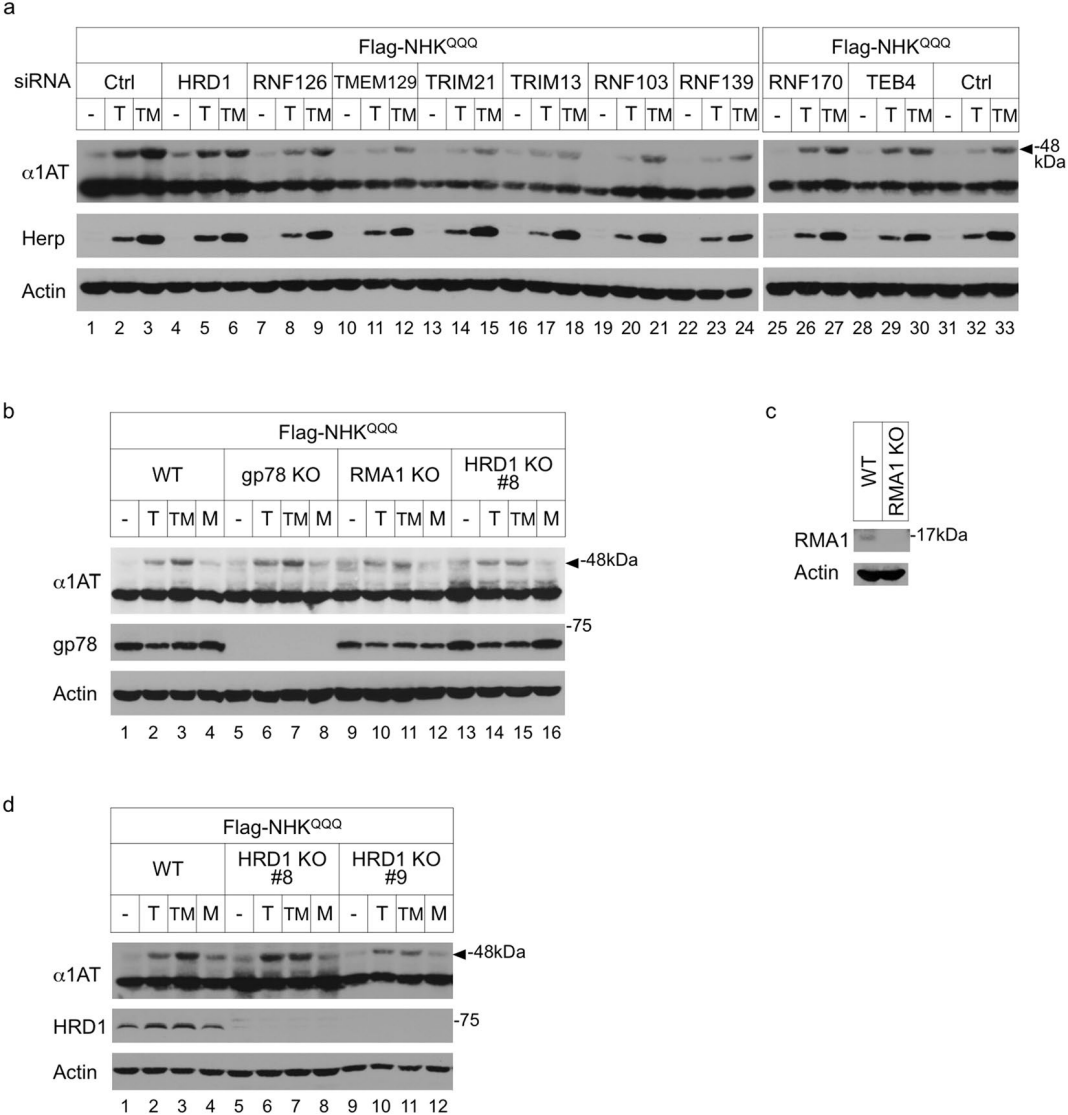
HRD1 is required for the proteasomal degradation of ERpQC substrates. (**a**) The effects of knockdown of ERAD-related and Bag6-related E3 ligases on the accumulation of ERpQC substrates. To clearly distinguish the size of ERpQC substrates from that of ERAD substrates, N-terminal Flag-tagged NHK^QQQ^ (Flag-NHK^QQQ^) was used as the ERpQC substrate. HEK293 cells were transfected with siRNA against control (Ctrl) or each E3 ligase and treated with the indicated combinations for 16 h after transfection with Flag-NHK^QQQ^-HA. Cell lysates were analyzed by IB. Arrowhead indicates ^S^NHK^QQQ^. T, 50 nM Tg; TM, 50 nM Tg and 200 nM MG132. (**b**–**d**) Deletion of HRD1 enhances the accumulation of ERpQC substrates in the absence of a proteasome inhibitor. WT, gp78 KO, RMA1 KO, or HRD1 KO (clone #8 or #9) HEK293 cells transfected with Flag-NHK^QQQ^-HA were treated with the indicated combinations and analyzed by IB. Deletion of HRD1 (**d**), gp78 (**b**), or RMA1 (**c**) was confirmed by IB using Ab against HRD1, gp78 or RMA1, respectively. Arrowheads indicate ^S^NHK^QQQ^. T, 50 nM Tg; TM, 50 nM Tg and 200 nM MG132. (**a**–**d**) Full-length blots are presented in Supplementary Fig. 5.

Signal peptide-uncleaved ERpQC substrates are degraded by the UPS. Although we previously demonstrated the roles of p97 and Bag6 in the degradation of ERpQC^5^, an E3 ligase that contributes to the ubiquitination of ERpQC substrates remains unknown. Theoretically, in cells lacking this ERpQC-related E3 ligase, proteasomal inhibition is expected to have no additional effect on the accumulation of ERpQC substrates during ER stress. Because rerouted ERpQC substrates attach to the ER membrane through binding with Derlin-1 in the presence of a proteasome inhibitor^5^, we hypothesized that ERpQC substrates are ubiquitinated by the E3 ligase on or near the ER membrane prior to proteasomal degradation. Therefore, we knocked down 7 ERAD-related transmembrane-type E3 ligases, including HRD1, TRIM13/RFP2, RNF103/Kf-1, RNF139/Trc8, RNF170, TEB4/March IV, and TMEM129^12,21^, and one ERAD-related cytosolic E3 ligase, TRIM21/Ro52^22^ (Fig. 2a), and we generated KO cells lacking 3 ERAD-related transmembrane-type E3 ligases, gp78, RMA1/RNF5, and HRD1, which are known to interact with Derlin-1^23,24^ (Fig. 2b,c). Since the depletion of ERAD-related E3 ligases could change ER conditions by affecting the retrotranslocation pathway, ER folding capacity and other degradation pathways such as autophagy, it is difficult to compare the amounts of accumulated substrates from different E3 ligase-deficient cells. Therefore, we examined the effect of MG132 on the accumulation of ERpQC substrates during ER stress in each E3 ligase-deficient cell line. The inhibition of proteasomal activity by MG132 increases the accumulation of ERpQC substrates in control (Ctrl) small interfering RNA (siRNA)-transfected HEK293 cells during ER stress (Fig. 2a, top panel, lanes 2 and 3, arrowhead). We measured the intensity of ^S^NHK^QQQ^ and NHK^QQQ^, and the relative amount of ^S^NHK^QQQ^ was calculated as relative to the total combined intensity of ^S^NHK^QQQ^ and NHK^QQQ^. The amount of ^S^NHK^QQQ^ in the Tg- and MG132-treated Ctrl cells was 1.49-fold that in the Tg-treated Ctrl cells (Fig. 2a, top panel, lanes 2 and 3, arrowhead). The knockdown experiments showed that in HRD1 siRNA-transfected cells, the Tg-induced accumulation of ^S^NHK^QQQ^ was minimally affected by treatment with MG132 (1.06-fold that in cells treated with Tg and without MG132; Fig. 2a, top panel, lanes 5 and 6, arrowhead). In other ERAD-related E3 ligase-knockdown or knockout cells, treatment with MG132 clearly enhanced the accumulation of ^S^NHK^QQQ^ during ER stress (Fig. 2a,b, arrowheads). The knockdown of RNF126 E3 ligase, which is required for the degradation of mislocalized ER-targeting proteins in coordination with Bag6^14^, also exhibited no effect on the MG132-induced accumulation of ^S^NHK^QQQ^ during ER stress (Fig. 2a, top panel, lanes 8 and 9, arrowhead). We confirmed that MG132 had no effect on the accumulation of ^S^NHK^QQQ^ during ER stress in 2 independent clones of HRD1 KO cells (Fig. 2d, top panel, lanes 6, 7, 10 and 11, arrowhead). We therefore propose that HRD1 is a candidate for the E3 ligase that promotes the ubiquitination of ^S^NHK^QQQ^ and contributes to the degradation of ERpQC substrates.

**Figure 3:**
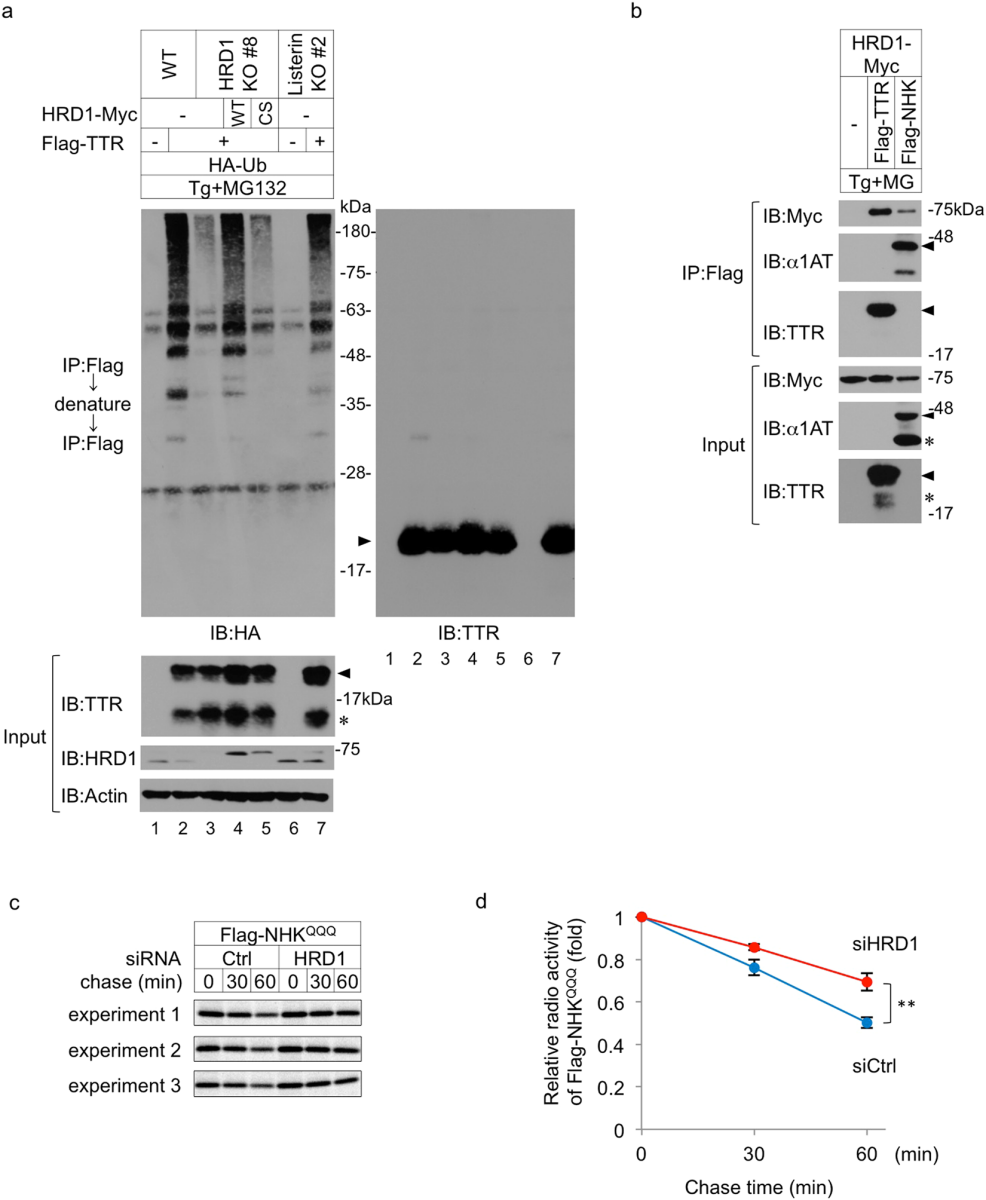
HRD1 is required for the ubiquitination of ERpQC substrates. (**a**) HRD1 contributes to the ubiquitination of ERpQC substrates through its E3 ligase activity. WT, HRD1 KO (clone #8) or Listerin KO (clone #2) HEK293 cells were treated with 50 nM Tg and 200 nM MG132 for 16 h after transfection with the indicated combinations. Flag-TTR-Myc was immunoprecipitated with an anti-Flag Ab affinity gel. After incubation with the denaturing buffer containing 1% SDS, Flag-TTR-Myc was re-immunoprecipitated with an anti-Flag Ab affinity gel and analyzed by IB with the indicated Abs. Arrowheads and asterisk indicate signal peptide-uncleaved TTR (^S^TTR) and signal peptide-cleaved TTR, respectively. CS, HRD1 (C291S/C329S)-Myc-His; Flag-TTR, Flag-TTR-Myc; HA-Ub, HA-Ubiquitin. (**b**) HRD1 interacts with ERpQC substrates. HEK293 cells were transfected with HRD1-Myc-His and Flag-TTR-HA or Flag-NHK^QQQ^-HA and treated with 50 nM Tg and 200 nM MG132. Cell lysates were analyzed by IP-IB using the indicated Abs. Arrowheads and asterisks indicate ERpQC substrates (^S^TTR and ^S^NHK^QQQ^) and ER translocated proteins (signal peptide-cleaved TTR and NHK^QQQ^), respectively. (**c** and **d**) The requirement of HRD1 for the degradation of ERpQC substrates. HEK293 cells were transfected with siRNA against Ctrl or HRD1 and Flag-NHK^QQQ^-HA and treated with 50 nM Tg for 16 h. Cells were pulse-labeled with [^35^S]-methionine/cysteine for 15 min and chased for the indicated time periods. Flag-NHK^QQQ^-HA was immunoprecipitated with an anti-Flag Ab affinity gel and analyzed by SDS-PAGE and autoradiography (**c**). The relative radioactivities in ^S^NHK^QQQ^ at different times of chase were calculated and shown as fold decreases relative to the intensity observed at 0 h chase. Values are expressed as the mean ± S.D. (**) P < 0.01; significance calculated by Student’s *t*-test (n = 3). (**a**–**c**) Full-length blots and gels are presented in Supplementary Fig. 5.

Next, to examine whether HRD1 ubiquitinates signal peptide-uncleaved ERpQC substrates, we performed a ubiquitination assay using N-terminal Flag-tagged transthyretin (TTR), which is another ERpQC substrate^5^. The deletion of HRD1, but not gp78 or RMA1 (Supplementary Fig. 2a,b), clearly reduced the poly-ubiquitination of signal peptide-uncleaved TTR (^S^TTR) (Fig. 3a, left top panel, lanes 2 and 3), and this effect was attenuated in cells transfected with HRD1 WT but not in cells transfected with the inactive E3 ligase HRD1 mutant (CS; Fig. 3a, left top panel, lanes 4 and 5). Listerin has been shown to recognize the 60 S ribosomal subunit-nascent chain complex and trigger nascent chain ubiquitination at the ribosome^25^. Therefore, we generated knockout cells to examine whether Listerin contributes to the ubiquitination of ERpQC substrates (Supplementary Fig. 2c). Listerin deletion had only a marginal effect on the ubiquitination of ^S^TTR (Fig. 3a, top panel, lanes 2 and 7). Taken together, our findings suggest that ERpQC substrates are mainly ubiquitinated by HRD1. We next investigated the recognition of ERpQC substrates by HRD1 and found that both signal peptide-uncleaved substrates ^S^NHK^QQQ^ and ^S^TTR interacted with HRD1 (Fig. 3b, top panel). Pulse-chase experiments revealed that HRD1 siRNA significantly delayed the degradation of ^S^NHK^QQQ^ compared with the effects of Ctrl siRNA (Fig. 3c,d). Similar results were observed in HRD1 KO cells, and the delay of ^S^NHK^QQQ^ degradation in HRD1 KO cells was restored by exogenously expressed HRD1 WT but not by HRD1 CS (Supplementary Fig. 2d,e). Collectively, these results strongly suggest that the degradation of ERpQC substrates is mainly mediated by the E3 ligase activity of HRD1.

### Association among the translocon, the rerouting factor Derlin-1, and the degradation factor HRD1

**Figure 4:**
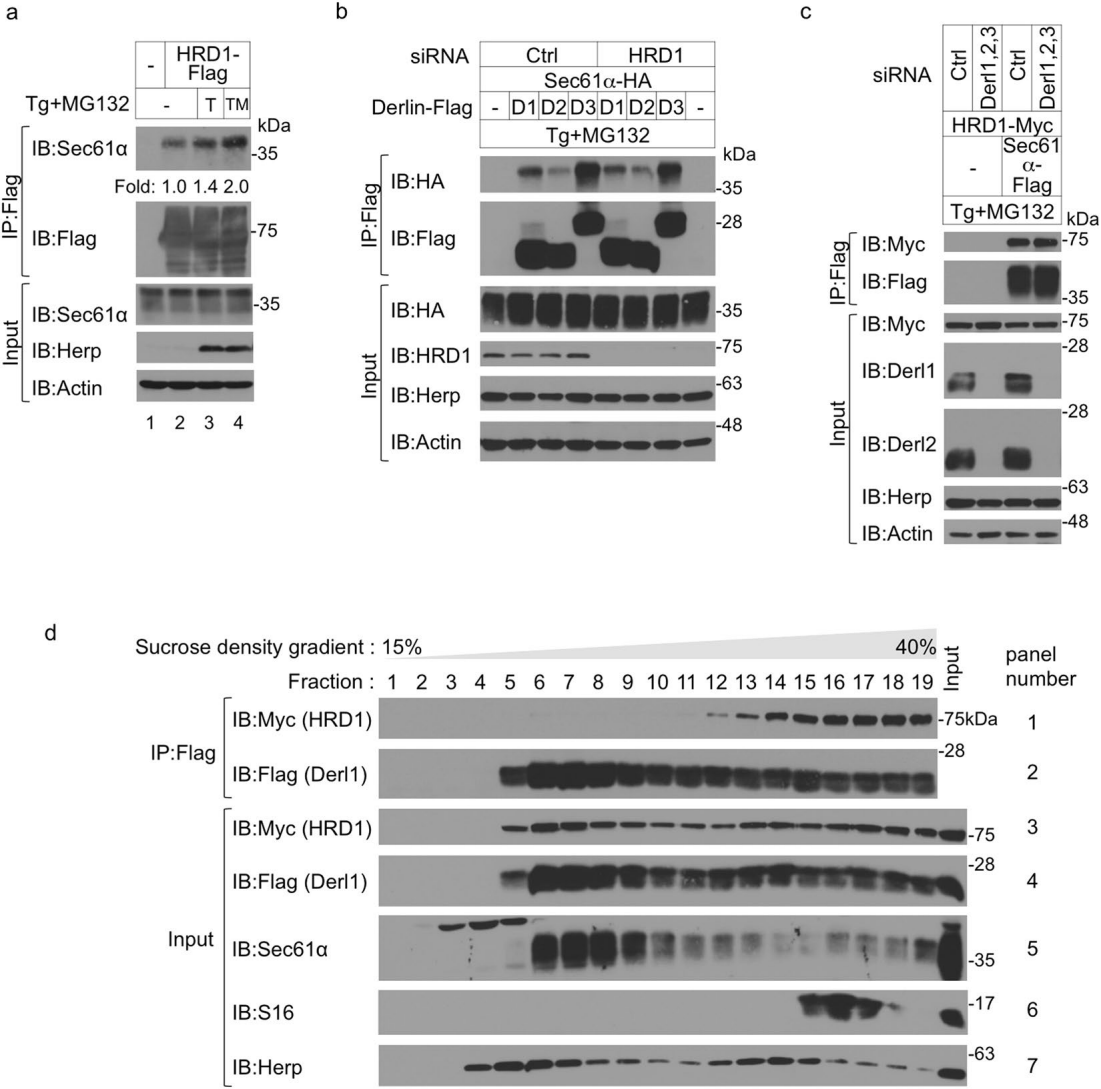
Association among the translocon component Sec61α, the rerouting factor Derlin-1, and the degradation factor HRD1. (**a**) HRD1 associates with Sec61α. HEK293 cells were transfected with HRD1-Flag and treated with the indicated combinations for 16 h. Cell lysates were analyzed by IP-IB with the indicated Abs. Sec61α co-immunoprecipitated with HRD1-Flag relative to the total amount of Sec61α was shown as fold increases compared with the unstressed condition. T, 50 nM Tg; TM, 50 nM Tg and 200 nM MG132. (**b**) HRD1 is not required for the interaction between Derlins and Sec61α. HEK293 cells were transfected with siRNA against Ctrl or HRD1, Sec61α-HA and Derlins-Flag and treated with 50 nM Tg and 200 nM MG132 for 16 h. Cell lysates were analyzed by IP-IB using indicated Abs. D1, Derlin-1; D2, Derlin-2; D3, Derlin-3. (**c**) Derlins are not required for the interaction between HRD1 and Sec61α. HEK293 cells were transfected with siRNAs against Ctrl or Derlin-1, -2 and -3, Sec61α-Flag and HRD1-Myc-His and treated with 50 nM Tg and 200 nM MG132 for 16 h. Cell lysates were analyzed by IP-IB using indicated Abs. (**d**) The high affinity of HRD1 for Derlin-1 in high-molecular-weight fractions HEK293 cells were treated with 50 nM Tg and 200 nM MG132 for 16 h after co-transfection with HRD1-Myc-His and Derlin-1-Flag. Cells were solubilized in 1% digitonin, and the soluble material was subjected to density gradient centrifugation in a 15–40% sucrose gradient. Each fraction (200 μl) was immunoprecipitated with an anti-Flag Ab affinity gel. Samples for IP and Input were analyzed by IB with the indicated Abs. (**a**–**d**) Full-length blots are presented in Supplementary Fig. 5.

We have previously demonstrated that rerouting factor Derlin family proteins interact with the Sec61α translocon and that ER stress enhances the recruitment of Derlin-1 and Derlin-3 to the translocon^5^. From the observation that HRD1 contributes to the degradation pathway in ERpQC, we hypothesized that this degradation factor may also associate with the translocon and assemble into the ERpQC complex with Derlins. As previously reported^23^, HRD1 associated with Derlin-1 (Supplementary Fig. 3a). This interaction was independent of the Derlin-1 CT, which is required for substrate rerouting, and the E3 ligase activity of HRD1 (Supplementary Fig. 3a,b). We next investigated the recruitment of HRD1 to the translocon. Exogenously expressed HRD1 associated with endogenous Sec61α (Fig. 4a, top panel, lane 2), and this association was increased by treatment with Tg (1.4-fold) or Tg and MG132 (2.0-fold; Fig. 4a, top panel, lanes 3 and 4). To further investigate the binding state of Derlin-1, HRD1, and Sec61α, we examined the effect of HRD1 or Derlin depletion on the recruitment of each to the translocon. Neither HRD1 siRNA nor Derlins siRNA had any effect on the interaction of these components with Sec61α (Fig. 4b,c). Taken together, these results suggest that Sec61α, Derlin-1, and HRD1 associate and form a complex on the ER membrane in response to ER stress.

To further characterize the complex formed by Sec61α, Derlin-1, and HRD1, cell extracts from WT HEK293 cells were subjected to sucrose density gradient centrifugation. During unstressed conditions, Derlin-1 sedimented in low- and middle-molecular-weight fractions (Supplementary Fig. 3c, 5th panel from the top, fractions 5–14), whereas HRD1 mostly sedimented in middle-molecular-weight fractions (Supplementary Fig. 3c, top panel, fractions 11–14). In ER-stressed cells, both HRD1 and Derlin-1 migrated at much higher-molecular-weight fractions (Supplementary Fig. 3c, 2nd and 6th panels, fractions 11–19). Among these fractions, middle-molecular-weight fractions containing HRD1, Derlin-1, and Herp seemed to constitute an ERAD complex as previously described^26–28^ (Supplementary Fig. 3c, 2nd, 6th and 10th panels, fractions 11–14). Moreover, treatment with a proteasome inhibitor in addition to the ER stressor increased the distribution of Derlin-1 in high-molecular-weight fractions containing rough ER marked with Sec61α and S16 ribosomal protein (Supplementary Fig. 3c, 7th, 15th and 23rd panels, fractions 14–19), suggesting that ER stress and proteasome inhibition induced the formation of a high-molecular-weight complex that may comprise the ERpQC complex including the translocon, Derlin-1, and HRD1. The ER stress-induced formation of the high-molecular-weight complex including the translocon and HRD1 was not attenuated by the deletion of Derlins (Supplementary Fig. 3d, 2nd, 8th, 12th, 14th, 18th and 22th panels, fractions 15–19). These results are consistent with the data on the Derlins-independent interaction of HRD1 with Sec61α (Fig. 4c). We finally investigated whether the rerouting factor Derlin-1 associates with the degradation factor HRD1 in the high-molecular-weight complex. Cell extracts from Derlin-1- and HRD1-cotransfected HEK293 cells treated with Tg and MG132 were subjected to sucrose density gradient centrifugation prior to a co-immunoprecipitation assay. Exogenously expressed Derlin-1 and HRD1 fractionated into broad peaks even after stimulation with an ER stressor and a proteasome inhibitor (Fig. 4d, 3rd and 4th panels, fractions 5–19), whereas the interaction between HRD1 and Derlin-1 was detected in the middle- and high-molecular-weight fractions (Fig. 4d, top panel, fractions 12–19). Interestingly, a higher affinity of HRD1 for Derlin-1 was observed in high-molecular-weight fractions containing rough ER marked with Sec61α and S16 (Fig. 4d, top, 5th and 6th panels, fractions 15–18). However, gp78 and RMA1, which can form an ERAD complex with Derlin-1^23,24^, associated with Derlin-1 in low- and middle-molecular-weight fractions but not in high-molecular-weight fractions (Supplementary Fig. 4a,b, top panels). Collectively, these observations suggest that the degradation factor HRD1 and the rerouting factor Derlin-1 form an ERpQC complex through association with the translocon.

## Discussion

**Figure 5:**
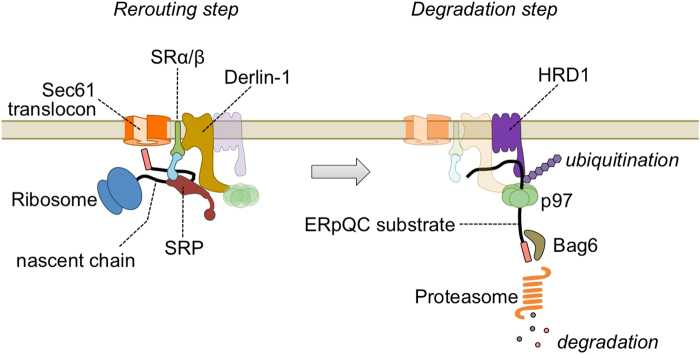
Schematic representation of the mechanism of ERpQC. Derlin-1 is recruited to the Sec61 translocon and SRP receptors (SRα/β) during ER stress. SRP54 on the ribosome nascent chain complex is trapped by the CT region of Derlin-1 and newly synthesized polypeptide emerged from ribosome is rerouted from the ER translocation pathway to the cytosolic degradation pathway (Rerouting step). The rerouted nascent chain (ERpQC substrate) is ubiquitinated by the E3 ligase HRD1, which forms a large ERpQC complex with the Sec61 translocon and Derlin-1 during ER stress, and effectively transported to the proteasome via the activities of p97 and Bag6 (Degradation step).

Our previous work has addressed the mechanism of ERpQC, which contributes to the maintenance of ER homeostasis. The ERpQC system is composed of a rerouting step and a degradation step^5^ (Fig. 5). ER stress promotes the rerouting of specific ER-targeting proteins to the cytosol without signal peptide cleavage. The rerouting of signal peptide-uncleaved ER proteins (ERpQC substrates) is mediated by Derlins, which interact with the SRP and SR during ER stress^5^. Although it remains unclear how Derlin-1 captures its substrates in the rerouting step, we have shown that the Derlin-1 CT (a.a. 207–251) is required for the recruitment of SRP54 and the capture of substrates (Figs 1 and 5). However, Derlin-1 ΔCT240 (a.a. 1–240), which cannot bind to p97, can still capture and reroute substrates^5^, suggesting that Derlin-1 CT (a.a. 207–251)-mediated rerouting is independent of the interaction of Derlin-1 with p97. Further investigation of Derlin-1 CT-binding proteins may clarify the precise mechanisms by which newly synthesized ER proteins are rerouted from the translocation pathway to the degradation pathway.

Rerouted ERpQC substrates are degraded by the UPS in a process that depends on the activities of p97 and Bag6^5^. Our findings revealed that HRD1 functions as the E3 ubiquitin ligase in ERpQC (Figs 2,3 and 5). HRD1 is a well-known component of the ERAD machinery that ubiquitinates retrotranslocated misfolded proteins^29,30^. Surprisingly, HRD1 was the only E3 ubiquitin ligase that targeted ERpQC substrates among a number of ERAD-related or ER transmembrane-type E3 ligases. In the case of ERAD, there are many types of substrates, e.g., proteins misfolded in the ER luminal domain (ERAD-L), the transmembrane domains (ERAD-M), or the cytosol (ERAD-C) and glycosylated or nonglycosylated proteins. This might be one of the reasons why a number of E3 ligases contribute to the ubiquitination of retrotranslocated proteins. The observations that marked UPR was not caused by a depletion of ERAD-related E3 ligase, including HRD1, may suggest that E3 ligases can compensate for each other (Fig. 2a). However, in the case of ERpQC, all substrates are newly synthesized polypeptides that emerged from the ribosome and were captured by Derlins without signal peptide cleavage or modification in the ER. Because the discrimination of these rerouted polypeptides is not necessary, one or a few E3 ligases may be sufficient to degrade ERpQC substrates. Moreover, the effective ubiquitination of ERpQC substrates by HRD1 may be convenient because of its association with the translocon and the rerouting factor Derlin-1.

Recent reports have shown that HRD1 functions as the core component of the retrotranslocon, which contributes to the movement of misfolded peptides through the ER membrane^31–33^. Whether one molecule of HRD1 ubiquitinates not only retrotranslocated ERAD substrates but also rerouted ERpQC substrates is unclear. Our sucrose density gradient experiments revealed that Derlin-1 fractionated into broad peaks in unstressed conditions, whereas some Derlin-1 shifted to higher fractions during ER stress (Supplementary Fig. 3c). Proteasome inhibition, in addition to ER stress, induced the further migration of Derlin-1 to high-molecular-weight fractions with rough ER marked with Sec61α and S16 ribosomal proteins (Supplementary Fig. 3c). From mass spectrometry-based proteomics analysis using Derlin-1-Flag, Derlin-2-Flag, or Derlin-3-Flag as bait, we identified several 40 S and 60 S ribosomal proteins, and some of these interactions were enhanced by ER stress (data not shown). It is unlikely that Derlin-associated ribosomal proteins function in ERAD. We also observed an interaction between Derlin-1 and HRD1 in the fractions containing S16 ribosomal protein (Fig. 4d, fractions 15–18). Collectively, we hypothesize that the direct or indirect interaction between Derlin-1 and translocon-associated RNC-SRP complex may contribute to the rerouting of ERpQC substrates from the ER translocation pathway to the HRD1-mediated degradation pathway (Fig. 5). Our findings suggest that the association among the translocon component, the rerouting factor Derlin-1, and the degradation factor HRD1 may be important for the efficient degradation of ERpQC substrates. Further investigation is necessary to clarify the precise mechanism by which Derlin-1 is mobilized to the large complex that includes the translocon during ER stress,

In conclusion, we report that HRD1 is an E3 ligase that contributes to the degradation of signal peptide-uncleaved substrates. HRD1 has been shown to retrotranslocate and ubiquitinate ER luminal unfolded or misfolded proteins, but here we have demonstrated a novel function of HRD1 as the E3 ubiquitin ligase of newly synthesized cytosolic polypeptides. Moreover, the ER stress-induced formation of a complex including HRD1, the translocon, and the rerouting factor Derlin-1 on the ER membrane could be important for the proper degradation of rerouted ER-targeting polypeptides to maintain ER homeostasis.

## Materials and Methods

### Cell culture

HEK293 cells were purchased from Invitrogen. *Derlin-1*, *HRD1*, *gp78*, *RMA1*, *Listerin*, and *Derlin-1*, *Derlin-2* and *Derlin-3* triple knockout HEK293 cells were generated in accordance with the relevant guidelines of University of Miyazaki. And all of the experimental protocols were approved by institutional guidelines of University of Miyazaki. All of cell types were cultured in Dulbecco’s modified Eagle’s medium containing 10% fetal bovine serum and penicillin-streptomycin solution.

### Gene editing

*Derlin-1* single knockout and *Derlin-1*, *Derlin-2* and *Derlin-3* triple knockout HEK293 cells were previously generated using the Clustered Regularly Interspaced Short Palindromic Repeats (CRISPR)/CRISPR-associated 9 (Cas9) system^5^. *HRD1*, *gp78*, *RMA1* and *Listerin* genes were edited in HEK293 cells by CRISPR/Cas9 system. The plasmids, pX335 (encoding a Cas9 D10A nickase) for knockout of the human *HRD1*, human *gp78*, or human *RMA1* gene, pX461 (encoding a Cas9 D10A nickase and the green fluorescent protein) for knockout of the human *Listerin* gene, and pX462 (encoding a Cas9 D10A nickase and the puromycin-resistant gene) for knockout of the human *Listerin* gene, were purchased from Addgene. Complementary oligonucleotides including single-guide RNAs (sgRNA) target sites (underlines) for the h*HRD1, hgp78, hRMA1, or hListerin* gene were as follows; hHRD1 target 1 5′-CACCGTGCGGAACATTGCCCTGGCC-3′ (forward) and 5′-AAACGGCCAGGGCAATGTTCCGCAC-3′ (reverse), hHRD1 target 2 5′-CACCGCGGCCAGCCTGGCGCTGAC-3′ (forward) and 5′-AAACGTCAGCGCCAGGCTGGCCGC-3′ (reverse); hgp78 target 1 5′-CACCGCCCGTGTAGGTGCGGAGGC-3′ (forward) and 5′-AAACGCCTCCGCACCTACACGGGC-3′ (reverse) and hgp78 target 2 5′-CACCGCTCAGCGGCCTGGCCCTGC-3′ (forward) and 5′-AAACGCAGGGCCAGGCCGCTGAGC-3′ (reverse); hRMA1 target 1 5′-CACCGAAACATATATTACATTCGA-3′ (forward) and 5′-AAACTCGAATGTAATATATGTTTC-3′ (reverse) and hRMA1 target 2 5′-CACCGGAGACTGCTCGGGAAGCTG-3′ (forward) and 5′-AAACCAGCTTCCCGAGCAGTCTCC-3′ (reverse); and hListerin target 1 5′-CACCTACTCTGAGCACTCAGACCC-3′ (forward) and 5′-AAACGGGTCTGAGTGCTCAGAGTA-3′ (reverse), and hListerin target 2 5′-CACCGCGAACTAAAGGGAACCTGA-3′ (forward) and 5′-AAACTCAGGTTCCCTTTAGTTCGC-3′ (reverse). Each knockout clone was established in HEK293 cells according to the previously described method^5^. All of methods to generate *Derlin-1*, *HRD1*, *gp78*, *RMA1*, *Listerin*, and *Derlin-1*, *Derlin-2* and *Derlin-3* triple knockout HEK293 cells were carried out in accordance with the relevant guidelines of University of Miyazaki. And all of the experimental protocols were approved by institutional guidelines of University of Miyazaki.

### Plasmids and transfection

Human Derlin-1-Flag, Derlin-1 ΔCT-Flag (a.a. 1–196, 1–206, 1–216 and 1–226), Venus-Derlin-1 CT-Flag (a.a. 197–251), Derlin-2-Flag, Derlin-3-Flag, 6Myc-SRP54, NHK^QQQ^, Flag-NHK^QQQ^-HA, Flag-TTR-Myc, Flag-TTR-HA, HRD1-Myc-His [wild-type (WT) and C291S/C329S (CS)], HRD1-Flag, 6Myc-RNA1, gp78-HA, HA-Ubiquitin, Sec61α-HA, and Sec61α-Flag, were constructed in pcDNA3.0 (Thermo Fisher Scientific) by polymerase chain reaction. Transfection was performed with Polyethylenimine-Max (Polysciences) according to the manufacturer’s instructions.

### Small interfering RNA knockdown

HEK293 cells were transfected with siRNA or control siRNA (Invitrogen) using Lipofectamine RNAiMAX reagent (Thermo Fisher Scientific). The sequences were as follows: HRD1-HSS149975 Stealth siRNA, GCCAAGAGACUGCCCUGCAACCACA and UGUGGUUGCAGGGCAGUCUCUUGGC; RNF126-HSS148069 Stealth siRNA, GCCAUGCAUGGUUUGUGGCGGAAGA and UCUUCCGCCACAAACCAUGCAUGGC; TMEM129-HSS150673 Stealth siRNA, GCGGAUUGACAAGUUUGCCACCGGU and ACCGGUGGCAAACUUGUCAAUCCGC; TRIM21-HSS110221 Stealth siRNA, GACAAUUUGGUUGUGGAACAAACAA and UUGUUUGUUCCACAACCAAAUUGUC; TRIM13-HSS145502 Stealth siRNA, CCUCAAGACACUGGCACAUUCAUUA and ™UAAUGAAUGUGCCAGUGUCUUGAGG; RNF103-HSS187898 Stealth siRNA, CCCUGUUUGCCGGUGGCCUUCUUAU and AUAAGAAGGCCACCGGCAAACAGGG; RNF139-HSS117458 Stealth siRNA, GAUACUUGUCCAAUGUGCCAUCAGA and UCUGAUGGCACAUUGGACAAGUAUC; RNF170-HSS130042 Stealth siRNA, GCCUGCAUUAUUGCUUACUGGCGAU and AUCGCCAGUAAGCAAUAAUGCAGGC; TEB4-HSS115753 Stealth siRNA, GGGUGGUAUCUUUAAAUACACUGUU and AACAGUGUAUUUAAAGAUACCACCC; Derlin-1-MSS228692 Stealth siRNA, AUAUAGUUGAAUCCAAGGAUAACCC and GGGUUAUCCUUGGAUUCAACUAUAU; Derlin-2-HSS121486 Stealth siRNA, AUAGACGAGCAUUAUUGUAAAGGCC and GGCCUUUACAAUAAUGCUCGUCUAU; Derlin-3-HSS150566 Stealth siRNA, UUGAAGAAGAAGCUGAAUCCCAGGG and CCCUGGGAUUCAGCUUCUUCUUCAA; and control siRNA, Negative Control Medium GC Duplex.

### Antibodies

Antibodies against α1AT (Dako, code no. A0012), TTR (DAKO, code no. A0002), HRD1 (Sigma-Aldrich, code no. H7915), gp78 (Cell Signaling Technology, code no. 9590), RMA1 (Santa Cruz Biotechnology, code no. sc-81716), Listerin (Abcam, code no. ab104375), Sec61α (Affinity BioReagents, code no. PA3–014), SRP54 (BD Biosciences, clone 30), ribosomal protein S16 (Santa Cruz Biotechnology, code no. sc-102087), Derlin-2 (MBL, code no. PM019), actin (Sigma-Aldrich, clone AC-40), Flag (Sigma-Aldrich, clone M2 and MBL, clone FLA-1), HA (Roche, clone 3F10 and Cell Signaling Technology, clone C29F4) and Myc (Calbiochem, clone 9E10) were purchased. The antibodies against Derlin-1 and Herp have been previously described^34,35^.

### Immunoprecipitation

Cells were lysed with lysis buffer (20 mM Tris-HCl pH 7.5, 150 mM NaCl, 5 mM EGTA and 1% Triton X-100) containing 5 μg/ml leupeptine. Cell lysates were immunoprecipitated with an anti-Flag M2 antibody (Ab) affinity gel (Sigma-Aldrich). The beads were washed with high-salt buffer (20 mM Tris-HCl pH 7.5, 500 mM NaCl, 5 mM EGTA and 1% Triton X-100) or low-salt buffer (20 mM Tris-HCl pH 7.5, 150 mM NaCl and 5 mM EGTA), resolved by SDS-PAGE and immunoblotted with antibodies. The proteins were detected with the ECL system. Aliquots of whole cell lysates were immunoblotted with antibodies.

### Immunoblotting

Cell lysates were resolved on SDS-PAGE and blotted onto PVDF membranes. After blocking with 5% skim milk in TBS-T (50 mM Tris-HCl pH 8.0, 150 mM NaCl and 0.05% Tween-20), the membranes were probed with antibodies. The proteins were detected with the ECL system.

### Ubiquitination assay

Transfected HEK293 cells were lysed on ice in lysis buffer (20 mM Tris-HCl pH 7.5, 150 mM NaCl, 5 mM EGTA and 1% Triton X-100) containing 20 mM *N*-ethylmaleimide (NEM) and 5 μg/ml leupeptine. Cell lysates were immunoprecipitated with an anti-Flag M2 antibody (Ab) affinity gel (Sigma-Aldrich). After washing with high-salt buffer (20 mM Tris-HCl pH 7.5, 500 mM NaCl, 5 mM EGTA, and 1% Triton X-100) and low-salt buffer (20 mM Tris-HCl pH 7.5, 150 mM NaCl and 5 mM EGTA), the beads were boiled in lysis buffer containing 1% SDS. The supernatant was diluted up to 50-fold with lysis buffer, re-immunoprecipitated with an anti-Flag M2 Ab affinity gel and analyzed by SDS-PAGE.

### Pulse-chase labeling assay

HEK293 cells transfected with siRNA against control (ctrl) or HRD1, and Flag-NHK^QQQ^-HA were labeled with [^35^S]-methionine/cysteine (EXPRE^35^S^35^S Protein Labeling Mix, PerkinElmer) in medium lacking methionine and cysteine for 15 min, washed with PBS and chased in medium containing excess methionine and cysteine. Cells were lysed with lysis buffer and immunoprecipitated with an anti-Flag M2 Ab affinity gel. Immunoprecipitated samples were resolved by SDS-PAGE and analyzed by autoradiography. The relative radioactivities in ^S^NHK^QQQ^ at different times of chase were calculated and shown as fold decreases relative to the intensity observed at 0 h chase. Values are expressed as the mean ± S.D. from three independent experiments. Statistical analysis was carried out by Student’s *t*-test. Statistical significance between two samples was determined by a P-value of less than 0.01. **P < 0.01.

### Sucrose gradient centrifugation

HEK293 cells were solubilized in 1% digitonin, and the soluble material was subjected to density gradient centrifugation in a 15–40% sucrose gradient. Centrifugation was performed at 100,000 × *g* at 4 °C for 16 h in an SW60 rotor (Beckman). Each fraction (200 μl) was collected from the top, analyzed by SDS-PAGE with/without IP using an anti-Flag M2 Ab affinity gel, and immunoblotting with antibodies.

## Electronic supplementary material

Supplementary Information
