# Supplementary material for: Molecular mechanism of ER stress-induced pre-emptive quality control involving association of the translocon, Derlin-1, and HRD1
Source: Sci Rep. 2018 May 9;8:7317. doi: 10.1038/s41598-018-25724-x (PMC5943263; doi:10.1038/s41598-018-25724-x)
Supplement: Supplementary file 1 — Supplementary Information [file 41598_2018_25724_MOESM1_ESM.pdf]

Supplementary information

Molecular mechanism of ER stress-induced pre-emptive quality control involving association of the translocon, Derlin-1, and HRD1

Hisae Kadowaki, Pasjan Satrimafitrah, Yasunari Takami, & Hideki Nishitoh

Supplementary Figure 1

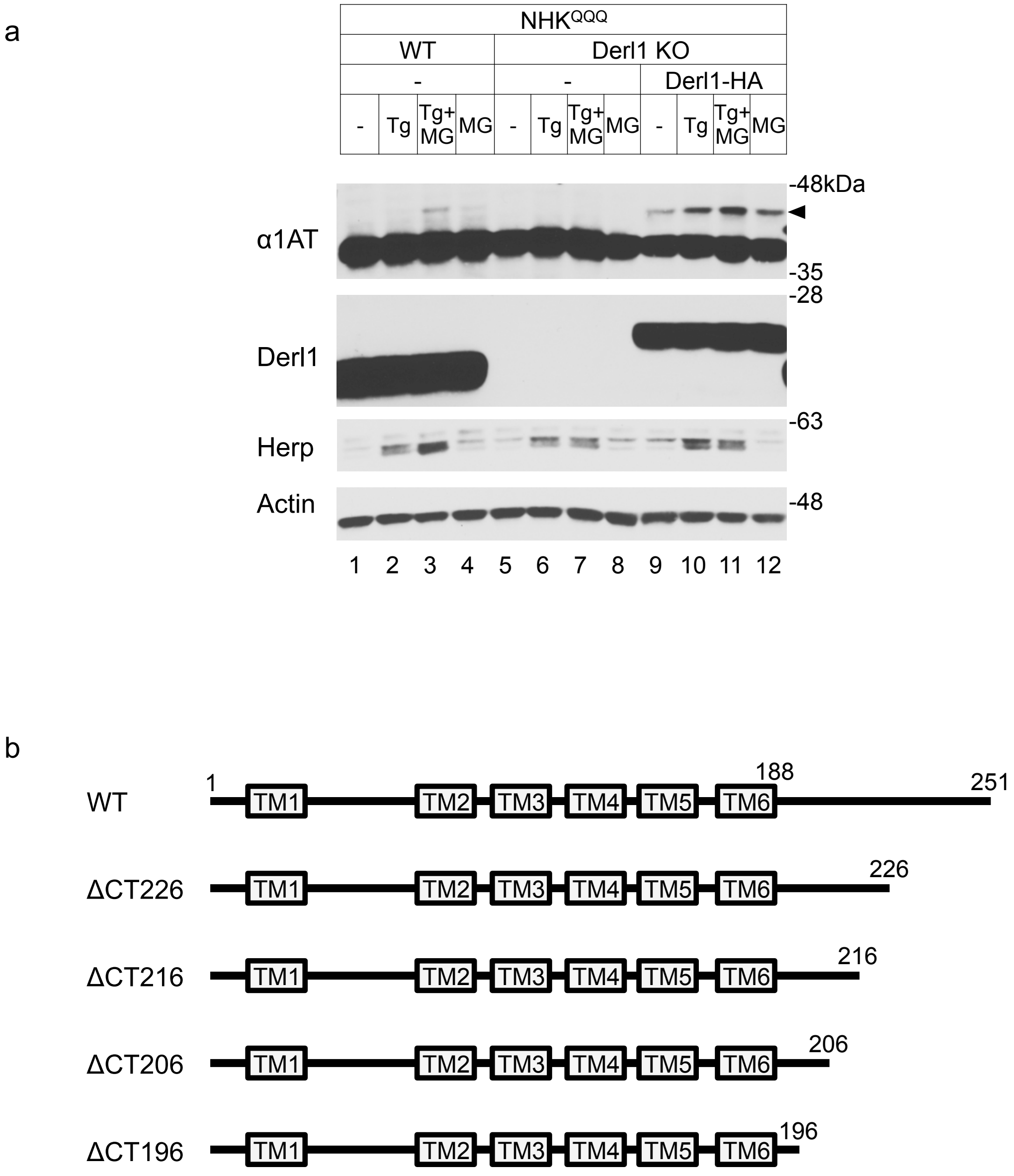

Supplementary Figure 1.

The requirement of Derlin-1 for ERpQC.

(a) WT or Derlin-1 knockout (Derl1 KO) HEK293 cells transfected NHK^{QQQ} with/without Derlin-1-HA (Derl1-HA) were treated with/without 50 nM thapsigargin (Tg), and/or 200 nM MG132 for 16 h. Cell lysates were analyzed by IB using indicated antibodies (Abs). MG, 200 nM MG132. Arrowhead indicates signal peptide-uncleaved NHK^{QQQ} ($^{S}NHK^{QQQ}$).

(b) Schematic representation of wild-type (WT) and various C-terminal-truncated mutant forms (ΔCT) of Derlin-1. TM indicates a transmembrane domain. ΔCT226, Derlin-1 (a.a. 1-226); ΔCT216, Derlin-1 (a.a. 1-216); ΔCT206, Derlin-1 (a.a. 1-206); ΔCT196, Derlin-1 (a.a. 1-196).

Supplementary Figure 2

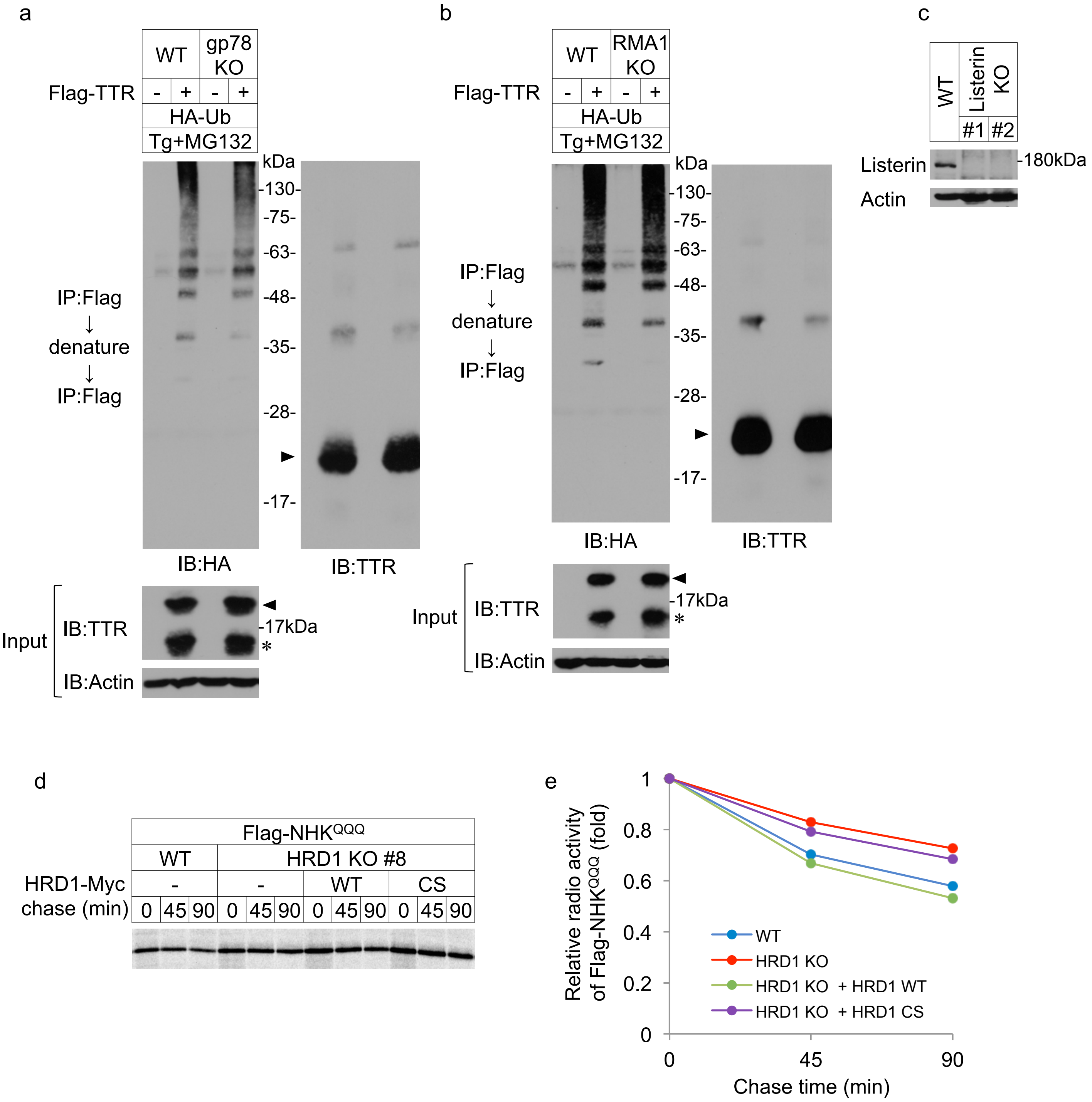

Supplementary Figure 2.

HRDI1, but not gp78 and RMA1, is indispensable for the proteasomal degradation of ERpQC substrates.

(a and b) WT, gp78 KO or RMA1 KO HEK293 cells were treated with 50 nM Tg and 200 nM MG132 for 16 h after transfection with the indicated combinations. Flag-TTR-Myc was immunoprecipitated and analyzed as in Figure 3a. Arrowheads and asterisks indicate signal peptide-uncleaved TTR (^{S}TTR) and signal peptide-cleaved TTR, respectively.

(c) Listerin KO clones (#1 and #2) were confirmed by IB using Ab against Listerin.

(d) WT or HRD1 KO HEK293 cells transfected with the indicated combinations were treated with 50 nM Tg for 16 h. Cells were pulse-labeled with [^{35}S]-methionine/cysteine for 15 min and chased for the indicated time periods. Flag-NHK^{QQQ}-HA was immunoprecipitated with an anti-Flag Ab affinity gel and analyzed by SDS-PAGE and autoradiography. CS, HRD1-Myc-His C291S/C329S.

(e). The relative radioactivities in ^{S}NHK^{QQQ} at different times of chase were calculated and shown as fold decreases relative to the intensity observed at 0 h chase.

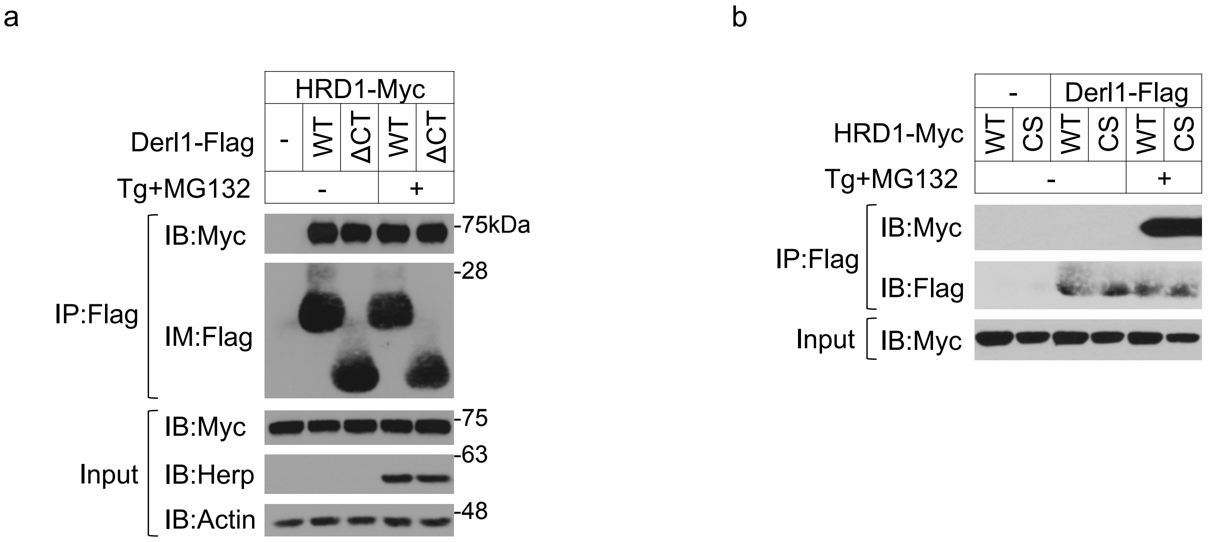

Supplementary Figure 3.

Sec61α, Derlin-1, and HRD1 associate and form a complex on the ER membrane in response to ER stress.

(a and b) HEK293 cells were treated with 50 nM Tg and 200 nM for 16 h after transfection with the indicated combinations. Cell lysates were analyzed by IP-IB using indicated Abs. ΔCT, Derlin-1 (a.a. 1-196); CS, HRD1-Myc-His C291S/C329S.

(c) HEK293 cells were treated with/without 50 nM Tg and 200 nM MG132 for 16 h. Cells were solubilized in 1% digitonin, and the soluble material was subjected to density gradient centrifugation in a 15% - 40% sucrose gradient. Each fraction (200 μl) was analyzed by IB with an anti-HRD1, anti-Derlin-1 (Derl1), anti-Herp, anti-Sec61α, anti-SRP54, or anti-S16 ribosomal protein Ab. T, 50 nM Tg; TM, 50 nM Tg and 200 nM MG132; MG, 200nM MG132.

(d) WT and Derlin-1, -2 and -3 triple KO (Derl TKO) HEK293 cells were treated with/without 50 nM Tg and 200 nM MG132 for 16 h. Cells were solubilized in 1% digitonin, and the soluble material was subjected to density gradient centrifugation in a 15% - 40% sucrose gradient. Each fraction (200 μl) was analyzed by IB with the indicated Abs.

Supplementary Figure 3

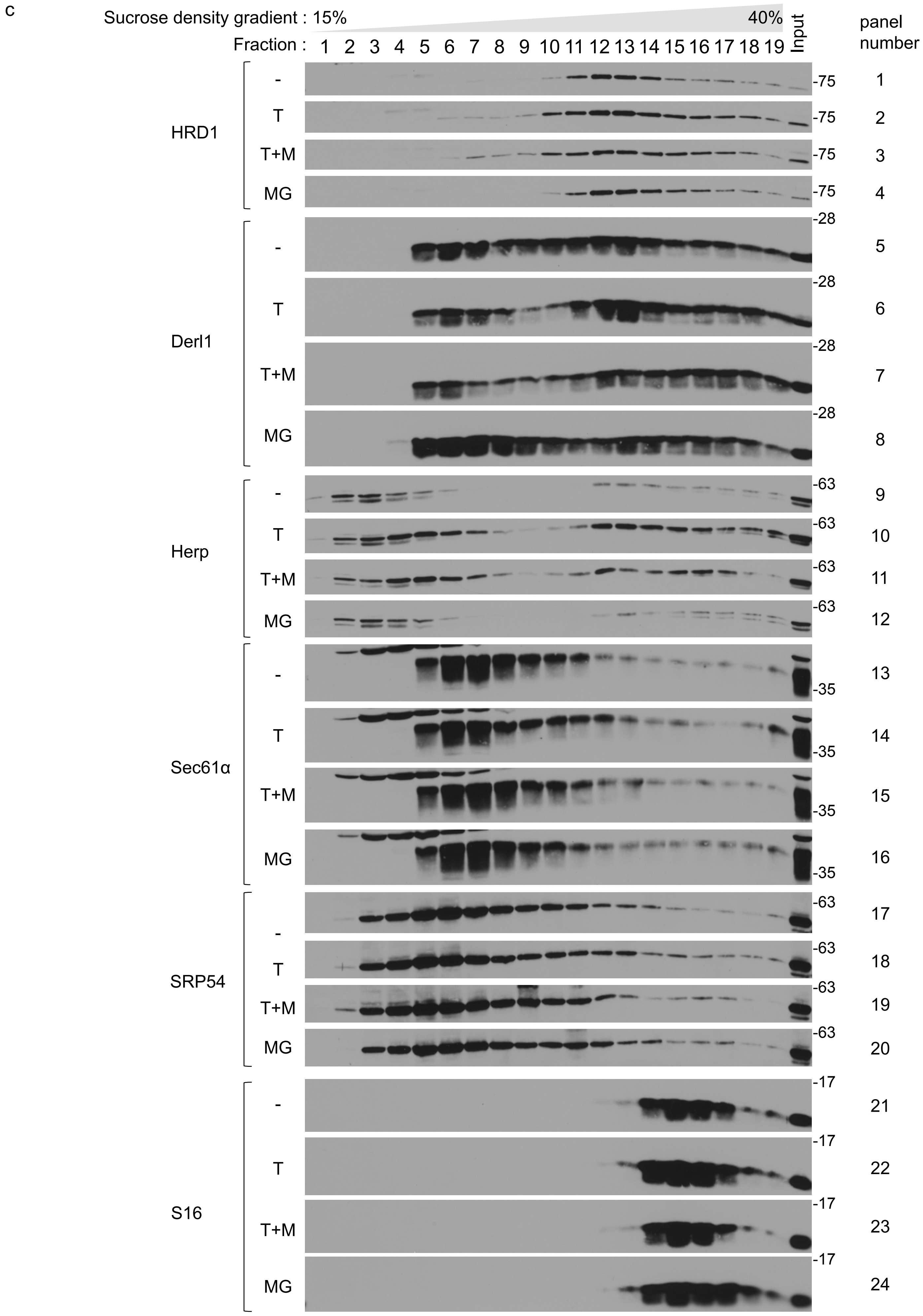

Supplementary Figure 3

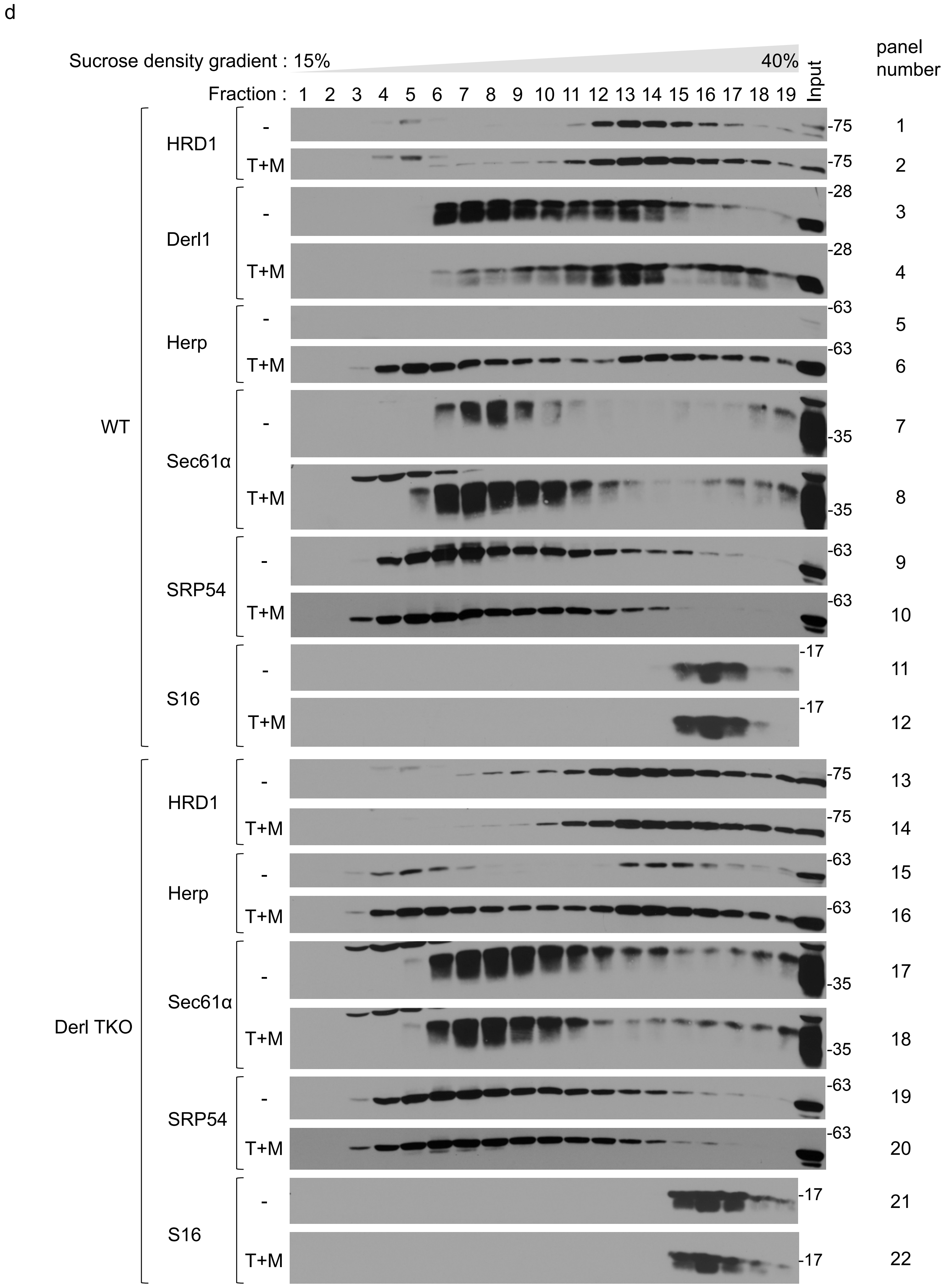

Supplementary Figure 4

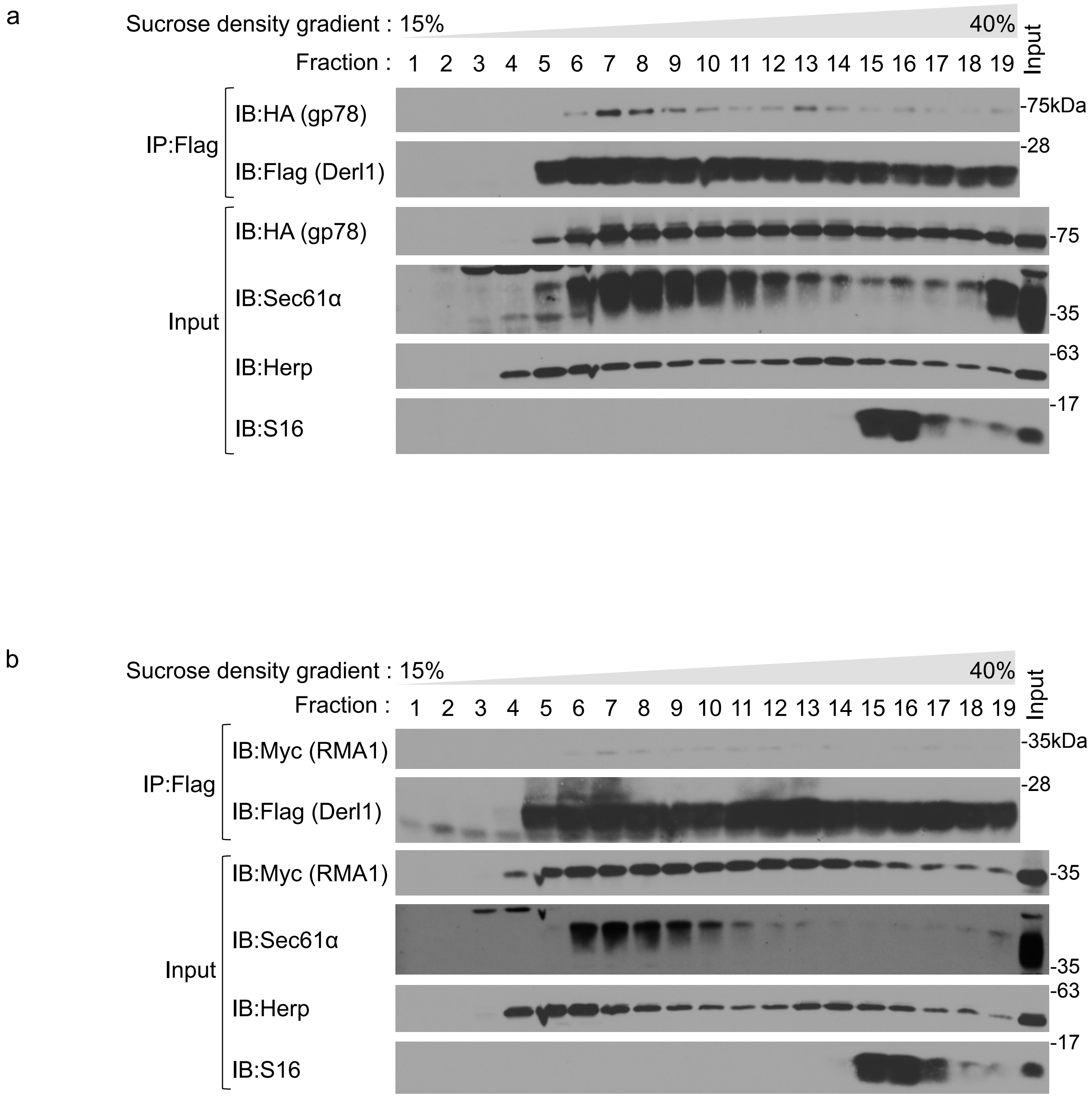

Supplementary Figure 4.

gp78 and RMA1 associate with Derlin-1 in a low- and middle-molecular-weight complex but not in a high-molecular-weight ERpQC complex.

(a and b) HEK293 cells were treated with 50 nM Tg and 200 nM MG132 for 16 h after co-transfection with gp78-HA (a) or 6Myc-RMA1 (b) and Derlin-1-Flag. Cells were solubilized and fractionated as in Figure 4d. Each fraction was immunoprecipitated with an anti-Flag Ab affinity gel. Samples for IP and Input were analyzed by IB with indicated Abs.

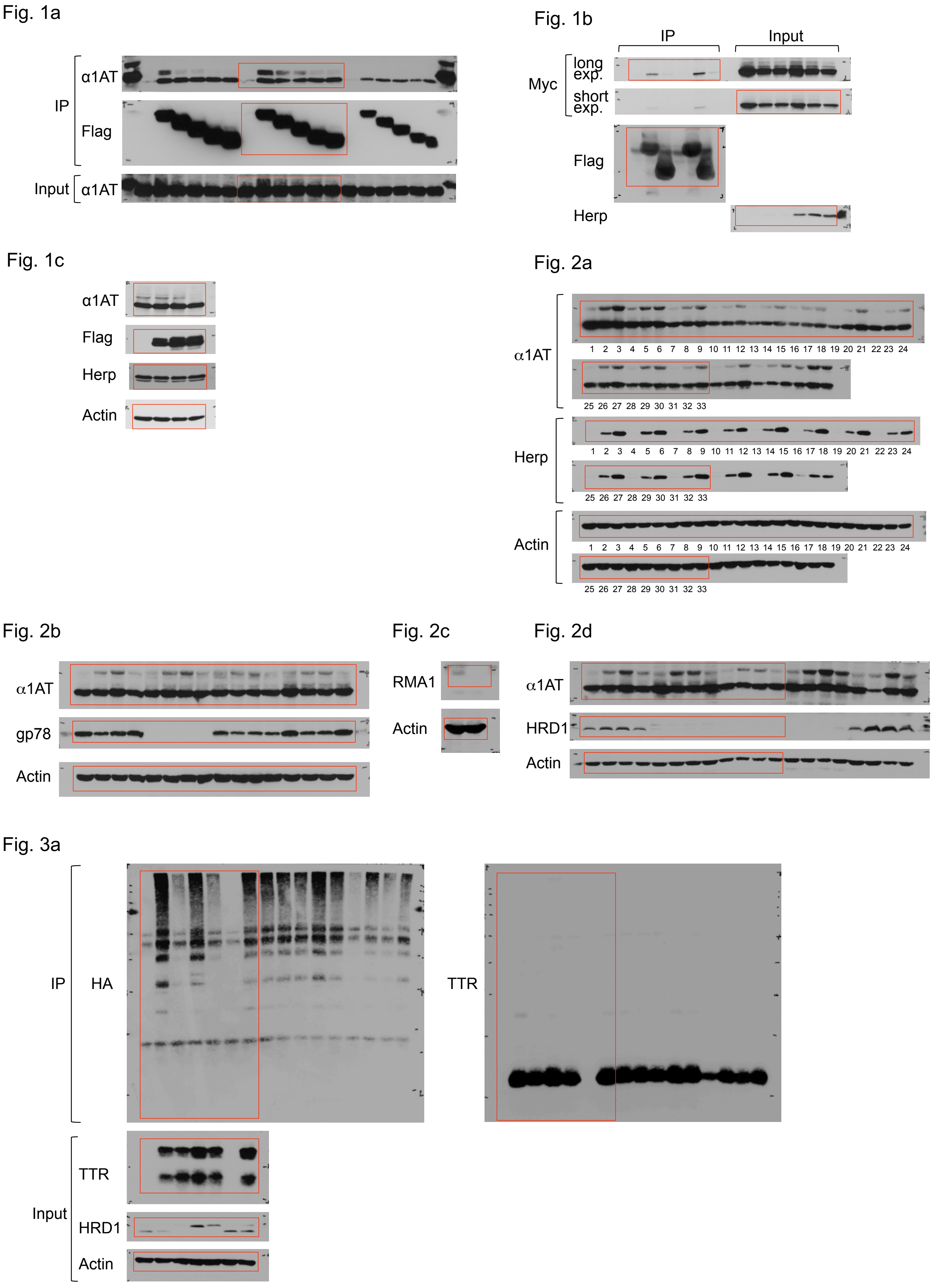

Supplementary Figure 5. Uncropped scans of blots and gels displayed in the main figures.

Fig. 3b

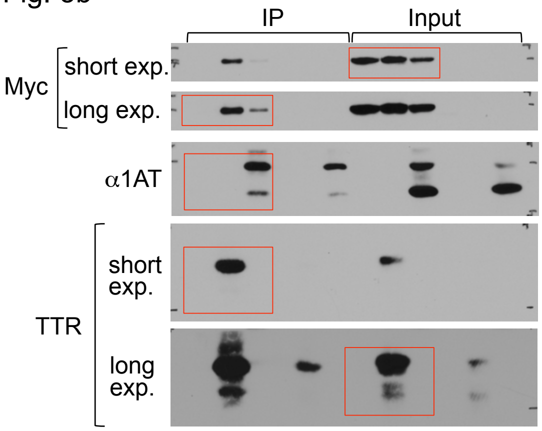

Fig. 3c

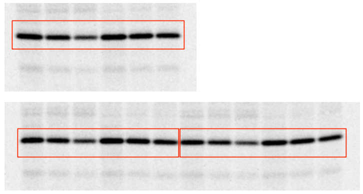

Fig. 4a

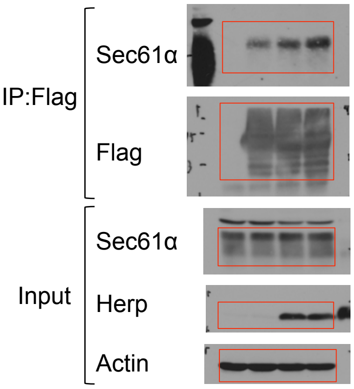

Fig. 4b

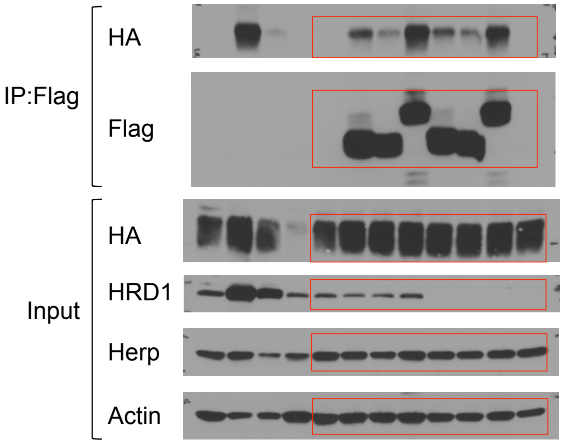

Fig. 4c

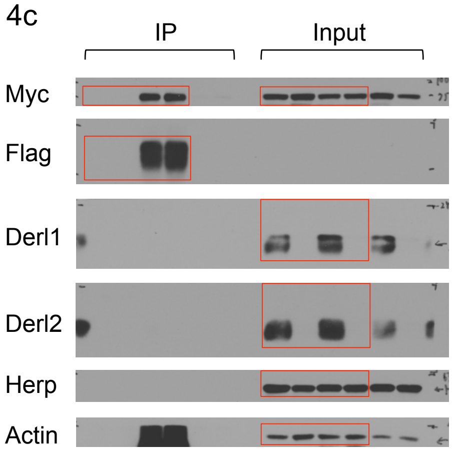

Fig. 4d

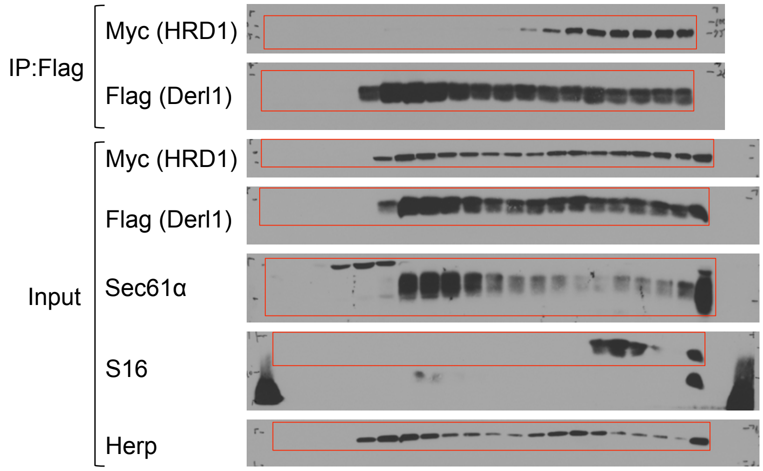

Supplementary Figure 5. Uncropped scans of blots and gels displayed in the main figures.
